# Use of Self-Reported Computerized Medical History Taking for Acute Chest Pain in the Emergency Department – the Clinical Expert Operating System Chest Pain Danderyd Study (CLEOS-CPDS): Prospective Cohort Study

**DOI:** 10.2196/25493

**Published:** 2021-04-27

**Authors:** Helge Brandberg, Carl Johan Sundberg, Jonas Spaak, Sabine Koch, David Zakim, Thomas Kahan

**Affiliations:** 1 Division of Cardiovascular Medicine Department of Clinical Sciences, Danderyd Hospital SE-182 88 Stockholm Sweden; 2 Medical Management Centre and Health Informatics Centre Department of Learning, Informatics, Management and Ethics Karolinska Institutet Stockholm Sweden; 3 Department of Physiology & Pharmacology Karolinska Institutet Stockholm Sweden

**Keywords:** chest pain, computerized history taking, coronary artery disease, eHealth, emergency department, health informatics, medical history, risk management

## Abstract

**Background:**

Chest pain is one of the most common chief complaints in emergency departments (EDs). Collecting an adequate medical history is challenging but essential in order to use recommended risk scores such as the HEART score (based on history, electrocardiogram, age, risk factors, and troponin). Self-reported computerized history taking (CHT) is a novel method to collect structured medical history data directly from the patient through a digital device. CHT is rarely used in clinical practice, and there is a lack of evidence for utility in an acute setting.

**Objective:**

This substudy of the Clinical Expert Operating System Chest Pain Danderyd Study (CLEOS-CPDS) aimed to evaluate whether patients with acute chest pain can interact effectively with CHT in the ED.

**Methods:**

Prospective cohort study on self-reported medical histories collected from acute chest pain patients using a CHT program on a tablet. Clinically stable patients aged 18 years and older with a chief complaint of chest pain, fluency in Swedish, and a nondiagnostic electrocardiogram or serum markers for acute coronary syndrome were eligible for inclusion. Patients unable to carry out an interview with CHT (eg, inadequate eyesight, confusion or agitation) were excluded. Effectiveness was assessed as the proportion of patients completing the interview and the time required in order to collect a medical history sufficient for cardiovascular risk stratification according to HEART score.

**Results:**

During 2017-2018, 500 participants were consecutively enrolled. The age and sex distribution (mean 54.3, SD 17.0 years; 213/500, 42.6% women) was similar to that of the general chest pain population (mean 57.5, SD 19.2 years; 49.6% women). Common reasons for noninclusion were language issues (182/1000, 18.2%), fatigue (158/1000, 15.8%), and inability to use a tablet (152/1000, 15.2%). Sufficient data to calculate HEART score were collected in 70.4% (352/500) of the patients. Key modules for chief complaint, cardiovascular history, and respiratory history were completed by 408 (81.6%), 339 (67.8%), and 291 (58.2%) of the 500 participants, respectively, while 148 (29.6%) completed the entire interview (in all 14 modules). Factors associated with completeness were age 18-69 years (all key modules: *P*s<.001), male sex (cardiovascular: *P*=.04), active workers (all key modules: *P*s<.005), not arriving by ambulance (chief complaint: *P*=.03; cardiovascular: *P*=.045), and ongoing chest pain (complete interview: *P*=.002). The median time to collect HEART score data was 23 (IQR 18-31) minutes and to complete an interview was 64 (IQR 53-77) minutes. The main reasons for discontinuing the interview prior to completion (n=352) were discharge from the ED (101, 28.7%) and tiredness (95, 27.0%).

**Conclusions:**

A majority of patients with acute chest pain can interact effectively with CHT on a tablet in the ED to provide sufficient data for risk stratification with a well-established risk score. The utility was somewhat lower in patients 70 years and older, in patients arriving by ambulance, and in patients without ongoing chest pain. Further studies are warranted to assess whether CHT can contribute to improved management and prognosis in this large patient group.

**Trial Registration:**

ClinicalTrials.gov NCT03439449; https://clinicaltrials.gov/ct2/show/NCT03439449

**International Registered Report Identifier (IRRID):**

RR2-10.1136/bmjopen-2019-031871

## Introduction

Chest pain is one of the most common chief complaints in emergency departments (EDs) worldwide [1,2]. Aside from electrocardiogram (ECG) and cardiac biomarkers, the medical history is regarded as central for management [3,4]. However, collecting an adequate medical history is a challenge for the physician due to limited time and is seldom done in a systematic, standardized way [5]. To improve chest pain management, emphasis has been put on developing new algorithms and advanced examinations [6-11].

Self-reported computerized history taking (CHT) is a method to collect a structured medical history by direct interaction between patients and a digital device. The concept of standardized history taking with structured paper questionnaires had already appeared in the 1940s [12]. The first software for CHT emerged in the 1960s [13]. Numerous CHT software programs have been developed and shown to collect more detailed data, as compared with conventional questionnaires [14]. CHT has the benefit of being reliable, as it never forgets to pose a question or diverges from what it is programmed to do [5]. As well, it can interpret the data instantly [15], which could aid the physician with complex information processing in a hectic environment (eg, triage in the ED). In several studies, CHT software collected more documented information than the physician (eg, in psychiatric history taking [16], outpatients with gastrointestinal symptoms [17] or dyslipidemia [18]). For the patient, highlighted benefits are that there is good acceptance of the software [14,16]; the patient is more likely to share sensitive information [14]; and consultation can be focused on identifying concerns and problems, rather than history taking [19]. The main disadvantages raised are irrelevant questioning, technical issues, and the programs' lack of empathy and inability to interpret body language [5,19].

Despite promising results, CHT is rarely used in clinical practice [5]. In 2007, a small feasibility study [20] including 64 patients showed that CHT was well accepted, that it collected an appropriate medical history of the various ED chief complaints, and that the concept could successfully be integrated with the process. However, there are only occasional studies on CHT in the acute cardiology setting or for ED patients with an acute complaint [20,21]. Indeed, the authors of a recent review for CHT in the management of cardiovascular disease concluded that there is a need to develop an evidence base for the use of CHT in this area of practice [22].

The overall aim of the Clinical Expert Operating System Chest Pain Danderyd Study (CLEOS-CPDS; ClinicalTrials.gov identifier: NCT03439449) is to determine the value of self-reported CHT for acute chest pain management [23]. This substudy is a utility study among the first 500 patients included, aimed to evaluate whether chest pain patients can effectively interact with CHT in the ED. Effectiveness was assessed as the proportion of patients completing the CHT interview and the time required to collect a medical history sufficient for cardiovascular risk stratification with an established risk score (ie, HEART [history, ECG, age, risk factors, and troponin] score; see below).

## Methods

### Setting

The CLEOS-CPDS study is an ongoing prospective cohort study recruiting consecutive patients presenting at the ED at Danderyd University Hospital (Stockholm, Sweden) from October 1, 2017, to December 31, 2023 (preliminary). The study has been described elsewhere [23] and has been approved by the Stockholm Regional Ethical Committee (now Swedish Ethical Review Authority) (reference number 2015/1955-1).

### Study Population

Clinically stable women and men (Rapid Emergency Triage and Treatment System [RETTS] level orange, yellow, green, and blue [24]) aged 18 years and older with a chief complaint of chest pain, fluency in Swedish, and a nondiagnostic first ECG or serum markers for acute coronary syndrome (ACS) were eligible for inclusion after providing informed consent. Patients unable to carry out an interview with CHT (eg, inadequate eyesight, confusion, or agitation) were excluded.

This study included the first 500 consecutive patients recruited (from October 1, 2017, to December 2, 2018). Danderyd University Hospital serves a population of approximately 600,000, and the ED had approximately 100,000 annual visits at the time of the study. The cardiology unit manages about 20% of the acute visits with about two-thirds walk-in patients and one-third patients arriving by ambulance. The average time spent in our ED for patients with a chief complaint of chest pain and RETTS level orange, yellow, green, or blue is 4 hours and 10 minutes, based on all 6920 visits from January through November 2018.

### Interventions

**Figure 1:**
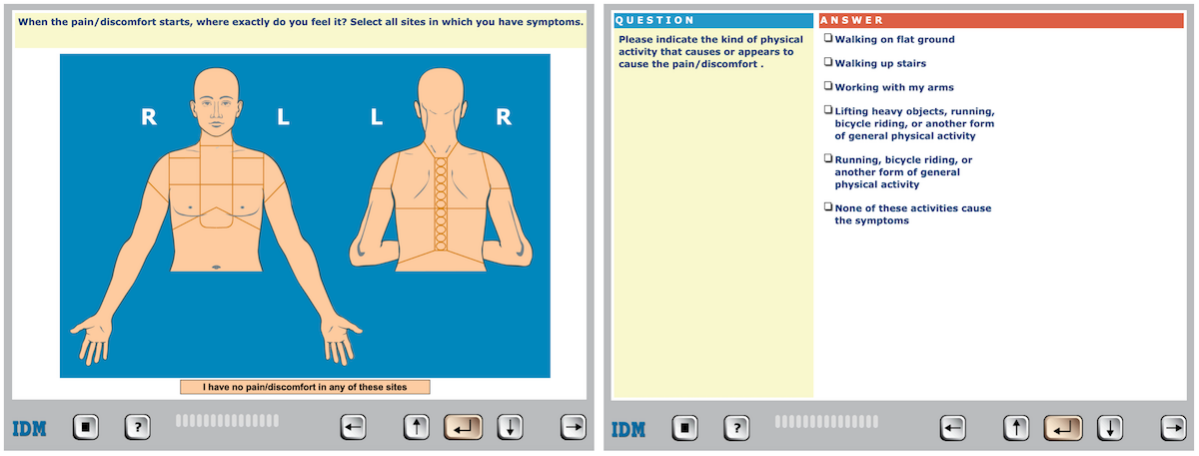
User interface of the Clinical Expert Operating System software with examples of a clickable image and multiple-choice question.

Self-reported CHT was conducted with the CLEOS software. CLEOS has been described in detail elsewhere [18,23,25]. In brief, the patient interacts with CLEOS on tablets (iPad, Apple Inc) by answering sets of questions for different medical modules starting with the patient’s chief complaint. Questions are mainly given in structured text format, such as yes/no or multiple-choice questions (one or many answers possible), but many questions display images, for instance asking the patient to click on an image of the upper body to indicate where pain is located (Figure 1).

The CLEOS software collects an in-depth history including demographics, present illness, organ systems review, medical history, prescription and over-the-counter medications, socioeconomic status, lifestyle, and family history. First, the software collects information on demographics and then reviews the major medical modules (Table 1). As this study specifically concerns chest pain management, questions regarding established risk factors for ACS were asked in the very first part of the interview. The interview is individually tailored by the software, where each question is determined on the basis of prior questions and a set of rules that interpret the clinical significance of prior answers. In total, the software has >17,000 decision nodes and can collect >40,000 data elements. The interview can be paused at any time when needed (eg, for physician encounter, lab test, or diagnostic imaging) and can be resumed whenever the patient has the opportunity.

**Table 1:** Consecutive order of medical modules in the interview.

Order	Module
1.	Chief complaint
2.	Cardiovascular
3.	Respiratory
4.	Immunology/rheumatology
5.	Endocrinology
6.	Gastroenterology/gastrointestinal surgery
7.	Hepatology
8.	Nephrology and urology
9.	Obstetrics and gynecology
10.	Neurology
11.	Hematology/oncology
12.	Mental health
13.	History of medical/surgical events
14.	Family history

### Data Collection

All patients presenting to the ED with a suspected cardiac condition were triaged by a cardiology consultant or senior resident (office hours) or by a trained nurse (out-of-office hours) using the triage protocol RETTS, where a targeted medical history is included. For chest pain patients, ECG and biomarkers were collected before admission to the cardiology unit or to the inpatient day-care unit. If further workup was not indicated, the patients could also be sent home directly from the triage. Less than 0.5% of the patients were sent home directly after triage. If there were signs of ST-elevation myocardial infarction on ECG or if the patient was clinically unstable, the patient was immediately admitted and not included in the study. For patients with a nondiagnostic first ECG, the physician in the cardiology unit or the inpatient day-care unit conducted a more thorough examination and standard history taking. For risk stratification, a combination of a modified HEART score [26], high-sensitivity cardiac troponin assays [10], and the 0/1 hour rule-in and rule-out algorithm [9] is recommended, according to regional guidelines. In the original HEART score, the History component is based on the physician’s subjective assessment. In this study, as well as recommended by regional guidelines, the traditional clinical classification of suspected anginal symptoms was used, that is, (1) central chest pain, (2) precipitated by physical or emotional exertion, and (3) relieved by rest or nitrates [27]. Depending on the number of characteristics met, the history was classified as highly (three characteristics met), moderately (two characteristics met), or slightly suspicious (none or one characteristic met) for angina pectoris.

The patients were offered the choice to participate in the CLEOS-CPDS study by a member of the research staff. Standardized oral and written information regarding the study was given, and the patients were given opportunity to ask questions before giving their informed consent by signing a consent form, all according to the procedures approved by the appropriate ethical committee (Multimedia Appendix 1). To ensure that the patient could navigate the CHT software, the research staff supervised the patient as the first page on demographics was answered. If the patient could not navigate the CHT software, the patient was not included. CHT was only performed during waiting times and did not interfere with routine work flow or care in the ED. CHT could occur before, after, or both before and after being seen by a physician, and the staff at the ED was not aware of the information collected by CLEOS at any time. The interview was discontinued either when it was fully completed, if the patient chose to stop for any reason, or if the patient was discharged from the ED or admitted for in-hospital evaluation and treatment. Reasons for not including patients who were considered eligible, the cause for noninclusion, and the cause of discontinuation were registered by the research staff.

Self-reported descriptive data, medical history data and demographics, and time stamps for completion of each question were collected from the CHT software. All pauses lasting more than 2 minutes were assumed to be interruptions in the interview and excluded when calculating durations. Data on arrival type, arrival time, and admission for the study population were extracted manually by research staff from the electronic health record (TakeCare, CompuGroup Medical Sweden AB). Demographic data and time spent in the ED for the general ED chest pain population during the study period were collected with QlikView, Version 12.10 (QlikTech International AB).

### Statistical Analysis

Study outcomes were (1) representativeness of the study population actually included (ie, age and gender of the study participants as compared with the general ED chest pain population), (2) the extent of interview completeness, overall and with regard to demographics, (3) the duration of interview segments, including completed modules, completed interview, and pauses, and (4) effectiveness, assessed as the proportion of patients completing the CHT interview to collect medical history sufficient for cardiovascular risk stratification with the established HEART score.

**Figure 2:**
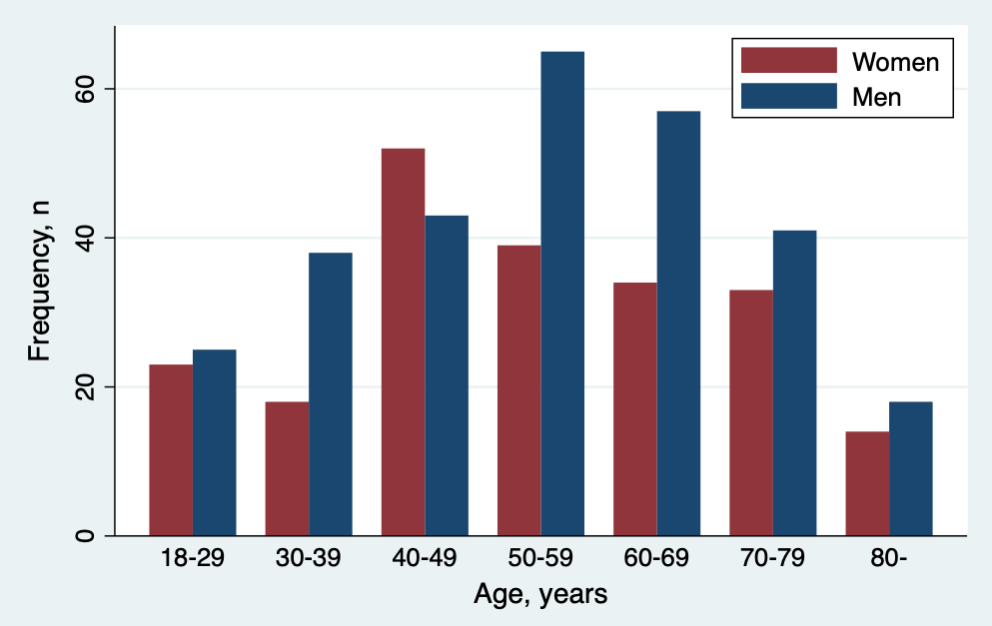
Age and sex distribution of the study population (n=500). Total number of patients in each age group—18-29: 48, 30-39: 56, 40-49: 95, 50-59: 104, 60-69: 91, 70-79: 74, 80 years or older: 32.

Descriptive statistics (mean values and standard deviations, median values and IQRs, or proportions, as appropriate) were used for patients’ baseline characteristics and to summarize completion and duration of key modules, HEART score data, and completed interview. Pearson *χ*^2^ tests (with 2 degrees of freedom) were used to compare the extent of completeness for binary variables. Wilcoxon rank sum tests were used to compare the median duration for completing the modules. Patients were stratified into 7 age groups (Figure 2), and time of arrival was grouped as morning (7 AM–noon), afternoon (noon–5 PM), evening (5 PM–10 PM), and night (10 PM–7 AM). To test for differences in completion for categorical variables with more than two groups (age groups, occupational status, and time of arrival), the Kruskal-Wallis test with a Dunn pairwise comparison with Bonferroni adjustments as post hoc analysis was used. All statistical analyses were performed using Stata, release 14 (StataCorp).

## Results

### Study Population and Their Characteristics

A total of 9532 patients presented at the ED with a chief complaint of chest pain during the entire study period. During the periods with research staff on duty (ie, when active inclusion was performed), 500 patients who met all inclusion criteria but no exclusion criteria were consecutively enrolled in the study. The study population (Table 2) had a similar age and sex distribution (mean 54.3, SD 17.0 years, and 213/500, 42.6% women, respectively) as compared with the general chest pain patient population (mean 57.5, SD 19.2 years, and 49.6% women). For patients who were considered eligible for the study but were not included, the most common causes were that they had language issues (182, 18.2%, mostly nonfluent in Swedish), that they felt too tired (158, 15.8%), or that they were unable to use a tablet (152, 15.2%) (Multimedia Appendix 2).

**Table 2:** Patient baseline characteristics (self-reported).^a^

Characteristic		Value	Responses
Age (years), mean (SD)		54.3 (16.7)	500
Women, n (%)		213 (42.6)	500
Body mass index (kg/m^2^), mean (SD)		26.4 (4.4)	500
Diabetes mellitus type 1 or 2, n (%)		27 (6.6)	412
Intake of lipid-lowering medication, n (%)		69 (19.6)	352
Hypertension, n (%)		163 (40.1)	406
Family history of coronary artery disease, n (%)		231 (57.0)	405
**Known coronary artery disease, n (%)**		74 (16.9)	437
	Angina pectoris	45 (10.3)	437
	History of myocardial infarction	47 (10.8)	437
	History of CABG^b^ or PCI^c^	53 (12.3)	432
No cardiovascular disease or diabetes, n (%)		322 (78.2)	412
Current smoker, n (%)		29 (7.0)	414
Previous smoker, n (%)		160 (38.6)	414
**Region of birth, n (%)**			500
	Nordic countries	415 (83.0)	
	Europe (outside the Nordic countries)	22 (4.4)	
	Outside Europe	63 (12.6)	
**Occupational status, n (%)**			500
	Active worker (employed, student)	320 (64.0)	
	Not at work (unemployed, on sick leave)	38 (7.6)	
	Retired	142 (28.4)	
Arrived at ED^d^ by ambulance, n (%)		92 (19.9)	463
**Arrival time, n (%)**			495
	Morning (7 AM–noon)	244 (49.3)	
	Afternoon (noon–5 PM)	180 (36.4)	
	Evening (5 PM–10 PM)	47 (9.5)	
	Night (10 PM–7 AM)	24 (4.8)	
Reporting any ongoing chest discomfort/pain, n (%)		264 (58.5)	451
Admitted (to the ward or day-care unit), n (%)		225 (46.0)	489

^a^Data from 500 patients are presented as mean values (SD) or n (%), as appropriate.

^b^CABG: coronary artery bypass grafting.

^c^PCI: percutaneous coronary intervention.

^d^ED: emergency department.

Age and sex distributions of the study population are presented in Figure 2. Self-reported patient characteristics and ED data are presented in Table 2. Mean age was about the same for women as for men (53.8 vs 54.6 years, respectively). Self-reported key clinical characteristics included known coronary artery disease (ie, angina pectoris, history of myocardial infarction, or history of coronary artery bypass grafting or percutaneous coronary intervention) in 16.9% (74/437), diabetes mellitus in 6.6% (27/412), hypertension in 40.1% (163/406), and lipid-lowering medication in 19.6% (69/352) (Table 2).

About one-fifth of the participants arrived by ambulance. Nearly half of the population presented to the ED during the morning (7 AM–noon) and about one-third in the afternoon (noon–5 PM). A majority of the patients (264/451, 58.5%) reported ongoing chest pain. Nearly half of the participants (225/489, 46.0%) were eventually admitted, either to a ward or an inpatient day-care unit (Table 2).

### Extent of Completeness

**Figure 3:**
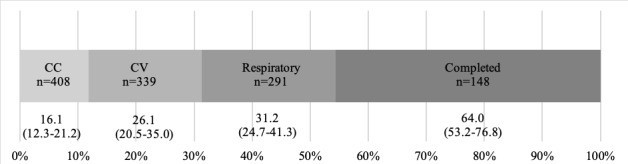
Median durations with IQRs (in minutes) for completed modules and complete interview, excluding pauses >2 minutes. CC: chief complaint module; Completed: completed interview; CV: cardiovascular module; Respiratory: respiratory module.

The number of participants who carried on with the interview decreased during the course of the interview (Table 3; Multimedia Appendix 3). Sufficient data to calculate HEART score (ie, clinical presentation and risk factors derived from a complete chief complaint module and the initial part of the cardiovascular module) were collected in 352 (70.4%) of the patients (Table 3). Of the 500 participants, the chief complaint (CC) module was completed by 408 (81.6%), the cardiovascular (CV) module by 339 (67.8%), and the Respiratory module by 291 (58.2%), while 148 (29.6%) completed the entire interview (Figure 3 and Table 3). Men completed the CV module and provided sufficient data to calculate the HEART score to a slightly greater extent than women (71.4% vs 62.9%, *P*=.04; 73.9% vs 65.7%, *P*=.049).

**Table 3:** Summary table for completion and duration of key modules, HEART score, and completed interview.^a,b^

Characteristic by sex			Chief complaint		Cardiovascular		Respiratory		Completed		HEART^c^ score
**Women (n=213)**											
	Completers, n (%)	170 (79.8)		134 (62.9)		117 (54.9)		54 (25.4)		140 (65.7)	
	Duration (min), median (IQR)	15 (12-20)		24 (20-35)		30 (24-39)		66 (53-75)		21 (18-30)	
	Duration (min), range	1-42		12-82		15-93		41-182		2-66	
**Men (n=287)**											
	Completers, n (%)	238 (82.9)		205 (71.4)		174 (60.6)		94 (32.8)		212 (73.9)	
	Duration (min), median (IQR)	17 (13-23)		27 (21-35)		32 (25-42)		63 (54-78)		24 (19-32)	
	Duration (min), range	1-77		11-88		14-97		32-121		2-85	
**All (n=500)**											
	Completers, n (%)	408 (81.6)		339 (67.8)		291 (58.2)		148 (29.6)		352 (70.4)	
	*P* value (completion)	.37		.04		.18		.07		.049	
	Duration (min), median (IQR)	16 (12-21)		26 (21-35)		31 (25-41)		64 (53-77)		23 (18-31)	
	Duration (min), range	1-77		11-88		14-97		32-182		2-85	
	*P* value (duration)	.12		.15		.43		.25		.08	

^a^Number (n) and proportions (%) of participants presented for completed modules, completed interview, and data sufficient for calculating HEART score before discontinuing the interview.

^b^No significant difference between sexes for duration, but significant difference for completion of cardiovascular module and complete HEART score.

^c^HEART: history, electrocardiogram, age, risk factors, and troponin.

**Figure 4:**
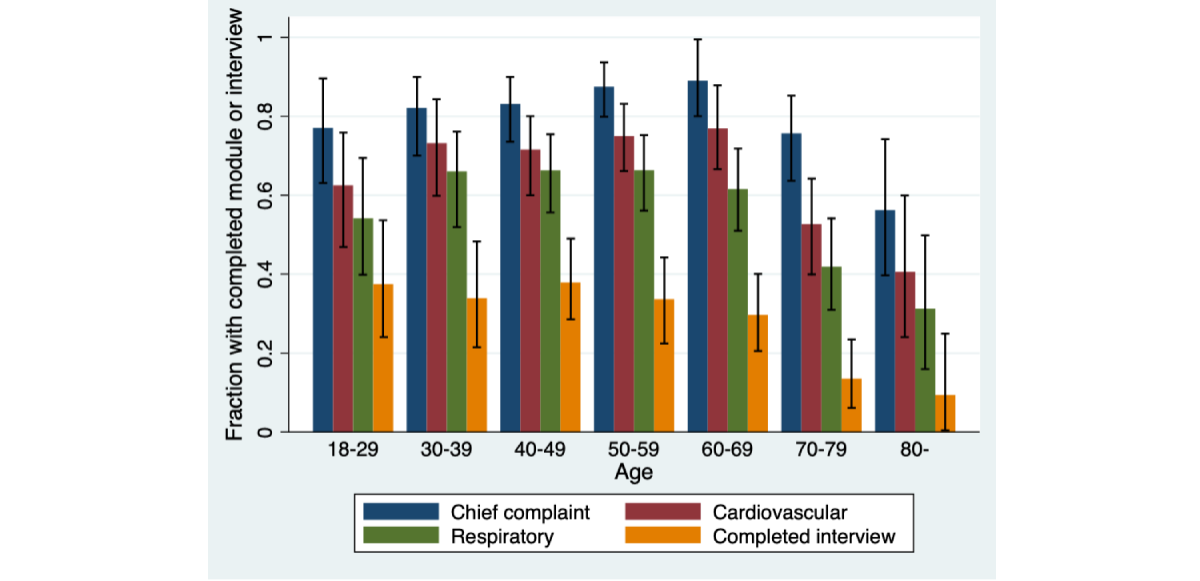
Fractions (with 95% CIs) of completed key modules and completed interviews, stratified by age.

**Figure 5:**
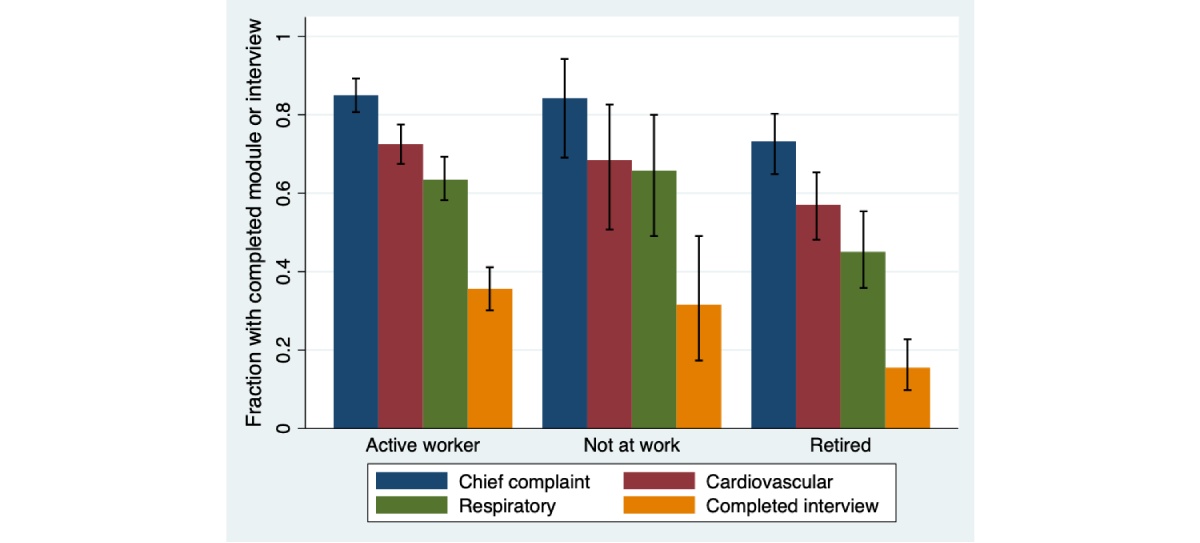
Fractions (with 95% CIs) of completed key modules and completed interviews, stratified by occupational status (320 active workers, 38 not at work, 142 retired). Active worker: employed or student; not at work: unemployed or on sick leave.

The proportion of the participants who completed the key modules in this context (CC, CV, and Respiratory modules) and the complete interview was lower in the age groups 70-79 years and 80 years or older, as compared with younger age groups (*P*s<.001 for all key modules and completed interview; Figure 4). No other significant differences in rates of completion were found between the age groups. Active workers completed the key modules and the complete interview more often than retired participants (CC: *P*=.004, CV: *P*=.002, Respiratory: *P*<.001, and complete interview: *P*<.001) (Figure 5). Patients not at work completed the Respiratory module to a slightly greater degree than retired participants (*P*=.03). No other significant differences were found by occupational status.

**Figure 6:**
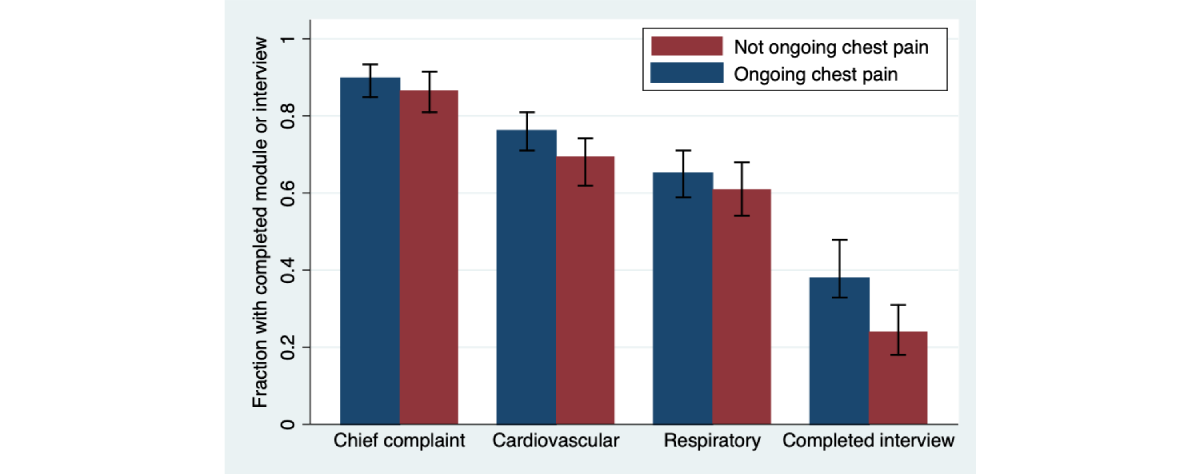
Fractions (with 95% CIs) of completed key modules and completed interviews, stratified by ongoing chest pain or not (264 with ongoing chest pain and 187 without).

Patients arriving by ambulance completed the CC and CV modules to a slightly lesser extent compared with those not arriving by ambulance (79% vs 88%, *P*=.03 and 63% vs 74%, *P*=.045, respectively). Participants who reported ongoing chest pain completed the interview to a greater extent than those not reporting ongoing chest pain (38% vs 24%, *P*=.002; Figure 6). No other significant differences in the completion of modules, for example, relation to the time of day the participant presented to the ED or by admission (hospital admission vs discharged home), were found.

### Duration of CHT Session

The median duration (excluding pauses longer than 2 minutes) to collect HEART score data was 23 (IQR 18-31) minutes, to complete the CC module 16 (IQR 12-21) minutes, to complete the CV module 26 (IQR 20-35) minutes, to complete the Respiratory module 31 (IQR 25-41) minutes, and to complete an entire interview 64 (IQR 53-77) minutes (Figure 3 and Table 3). No difference for duration by sex was found. The number and proportions of patients who ended the interview within a certain time and mean pauses stratified by interview duration are presented as supplementary material (Multimedia Appendices 4 and 5).

In the group of 352 participants who did not complete the full interview, the main reasons for discontinuing were discharge from ED (101, 28.7%) and that the participant felt tired (95, 27.0%) (Table 4). When comparing the age groups 18-69 years (n=259) and ≥70 years (n=93) to participants of all ages, discharge from ED was more frequent in the first group (28.7% vs 12.9%, *P*<.001), and difficulty using the tablet was reported more often in the second group (4.5% vs 14.0%, *P*<.001) (Table 4).

**Table 4:** Reasons for discontinuing the interview.^a^

Reasons	Age groups (years), n (%)			*P* value
	All	18-69	≥70	
Discharge from ED^b^	101 (28.7)	89 (34.4)	12 (12.9)	<.001
Tired	95 (27.0)	69 (26.6)	26 (28.0)	.81
Missing/not stated	61 (17.3)	48 (18.5)	13 (14.0)	.32
Admission/transfer	22 (6.3)	13 (5.0)	9 (9.7)	.11
Difficulty to use tablet	16 (4.5)	3 (1.2)	13 (14.0)	<.001
Clinical examination	15 (4.3)	11 (4.2)	4 (4.3)	.98
Technical issues	11 (3.1)	8 (3.1)	3 (3.2)	.94
End of research staff work shift	10 (2.8)	6 (2.3)	4 (4.3)	.32
Perceived not relevant/too many questions	9 (2.6)	5 (1.9)	4 (4.3)	.21
Acute medical condition/measure	7 (2.0)	3 (1.2)	4 (4.3)	.06
Other	5 (1.4)	4 (1.5)	1 (1.1)	.75

^a^Number (n) and proportions (%) of all 352 participants who did not complete the full interview, according to age group.

^b^ED: emergency department.

## Discussion

### Principal Results

Although the utility of CHT has been studied in primary care settings [28,29] and general acute settings [20,21], this appears to be one of the first studies of CHT in an acute cardiology setting. We show that a majority (70.4%) of acute chest pain patients can interact with CHT to collect medical history adequately to provide a HEART score for chest pain management. Given the large proportion of people presenting to the ED with chest pain, our results suggest that CHT could potentially contribute to safer management with improved risk stratification in this patient group, particularly during periods with high workload and crowding, which are associated with worse outcomes [30]. This could eventually reduce unnecessary, expensive, and potentially risky examinations. However, our study only shows the utility of this specific strategy for patient interview. Further studies are needed to validate the information provided by the CLEOS CHT program against information in the electronic health record obtained by an interview performed by a physician and to evaluate the results to prospective outcome data.

The interview was arranged so that the most important factors for assessing chest pain were asked in the very first part, in order to collect medical history sufficient for cardiovascular risk stratification in the ED setting. Subsequently, data was collected for all organ systems with, in the CLEOS developers' opinion, lesser significance for the assessment of chest pain the longer the interview went on. As expected, the proportion of patients who continued with the interview decreased the longer it went on. More importantly, however, the median duration for sufficient data to calculate the HEART score was only 23 minutes. This is comparable to the reported time for taking a standard history in an acute setting [31,32]. However, the CHT can make use of the waiting time in the ED and provide the patient with time to think through their answers more carefully, which could add to more reliable answers and improved diagnostic results.

Premature discontinuations were mainly due to patients being sent home or getting too tired to continue the interview. Only 30% went through a complete interview, with a median duration of 64 minutes, which is longer than a standard interview by a physician in an ED setting [31,32]. However, it is important to consider the context and the intention of data collection when assessing the duration of the interview. Reaching a fully completed interview is of importance to identify an unclear diagnosis or for research purposes, but for risk stratification in acute chest pain patients, it is more important to rapidly populate the elements included in an established risk score. Of note, in the current study of patients with mostly low-intermediate risk (ie, RETTS level orange, yellow, green, and blue), median time spent in the ED was about 4 hours. Thus, the somewhat longer time for the CHT would not prolong the time spent in the ED, as compared to using a standard history taking by the attending physician. Nevertheless, it would be of benefit in future development of CHT if the extent of an interview could be adjusted to the context of the visit and medical urgency.

The extent of completion was lower in the age group 70 years and older, a finding not previously observed in the few studies available [15,17,18,20]. Our data suggests that this was due to difficulties using tablets and to somewhat more frequent hospitalizations than in the younger (18-64 years) age group (Table 4). Patients reporting ongoing chest pain also completed the interview to a slightly greater extent, which raises the question of whether one is more inclined to complete the interview due to concerns about a present complaint. As well, the group of patients arriving by ambulance completed two key modules of the interview (CC and CV) to a lesser extent than walk-in patients, possibly due to a larger proportion of older people in this group.

There are some strengths of this study. First, this is a large sample of patients from a study population representative of a general chest pain population, CLEOS-CPDS, with a prospective cohort study design and a published study protocol. Second, a generic layout of the CHT software may allow the results to be generalized to other complaints and care settings. Finally, this is an academically initiated and driven study where the CHT software is owned by a public university. There are no commercial interests within the research project.

There are also important limitations to this study. First, although patients were recruited consecutively, this occurred mainly during office hours and evenings (due to research staff working hours) and when a sufficient number of tablets was available. This entails a risk of selection bias, and the results may not apply to patients presenting to the ED at other times of the day. However, the proportion of chest pain patients arriving at night was small, and the demographics were similar to the total ED chest pain population. Second, there may also be confounding by a selection of patients with good tablet skills. This potential confounding warrant further study. As well, a number of patients were not eligible; patients with language difficulties or inability to carry out CHT on the tablet were not included. These groups accounted for 18.2% and 15.8% of patients who were asked to participate but did not. However, this compares to results found by others, where no complete basic medical history could be obtained in the ED setting for 25% of the patients [31]. Thus, it is important in future CHT implementations to identify these patient groups and their characteristics, so they can be offered standard history taking. In addition, developing simpler and more user-friendly software in these patient groups is needed. Third, patients sent home directly from the physician triage (potentially healthier and younger) were not eligible for the study. Many EDs do not have physician triage, and the assessment may vary between physicians [33]. This may affect the generalizability of the study. However, a negligible fraction of patients were sent home directly from the medical triage, and we consider it unlikely to have affected the study results. Finally, our results may not be applied for critically ill patients, who should be seen by a physician immediately according to the RETTS triage protocol, as the CHT was not intended for use in these patients.

### Conclusions

A majority of acute chest pain patients can interact effectively with CHT on a tablet in the ED to provide sufficient data for risk stratification with a well-established risk score (ie, HEART score). The utility was somewhat lower in patients 70 years and older, in patients arriving by ambulance, and in patients without ongoing chest pain. Further studies are warranted to assess whether CHT can contribute to improved management and better risk stratification for one of the most common chief complaints in the ED.

## Appendix

Multimedia Appendix 1 Patient information sheet and consent form according to the standards of GCP-ICH applied in Sweden. These documents are reviewed and approved by the Stockholm Regional Ethical Committee (reference number 2015/1955-1).

Multimedia Appendix 2 Reasons for not participating in the study (n=1000).

Multimedia Appendix 3 Number and proportion of participants who ended the interview within a certain time.

Multimedia Appendix 4 Fractions of completed modules for all participants. Only females could answer the Obstetrics/Gynecology module. Gastro: gastrointestinal.

Multimedia Appendix 5 Interview duration and number of pauses >2 minutes with mean pause duration stratified by interview duration.

## Abbreviations

ACS: acute coronary syndrome
CC: chief complaint
CHT: computerized history taking
CLEOS: Clinical Expert Operating System
CLEOS-CPDS: CLEOS Chest Pain Danderyd Study
CV: cardiovascular
ED: emergency department
ECG: electrocardiogram
HEART: history, electrocardiogram, age, risk factors, and troponin
RETTS: Rapid Emergency Triage and Treatment System
