# Supplementary material for: Use of Self-Reported Computerized Medical History Taking for Acute Chest Pain in the Emergency Department – the Clinical Expert Operating System Chest Pain Danderyd Study (CLEOS-CPDS): Prospective Cohort Study
Source: J Med Internet Res. 2021 Apr 27;23(4):e25493. doi: 10.2196/25493 (PMC8114166; doi:10.2196/25493)
Supplement: Multimedia Appendix 1 [file jmir_v23i4e25493_app1.doc]

N.B. Patient information sheet and consent form translated to English.

Please see below for the Swedish version used in this study.

# CLEOS-CPDS: Patient Information Sheet

**Computerized history taking for management of chest pain patients in the emergency department**

**Background and purpose**

In Sweden, more than 200,000 people/year seek emergency care due to chest pain. The cause of chest pain can be serious, but is often completely benign. To determine this, examination, sampling and a medical interview are required. Despite this, in some cases it can be difficult to rule out a serious underlying cause and further examinations or observation in the hospital may be required. In addition, previous studies have shown that patients who seek emergency care for chest pain, but who do not have a serious illness, seek medical care often and are on sick leave to a greater extent than healthy.

The main purpose of this study is to investigate whether an in-depth interview via a tablet (iPad) can provide a better basis for the physician than a traditional medical interview in the emergency department, to determine whether the chest pain is benign or not. We also want to investigate whether such an interview can predict morbidity among patients who seek the emergency department due to chest pain, but who do not have a serious illness. In the future, we hope to be able to provide better treatment for these patients as well.

**Request for participation**

You are asked to participate in the study because you seek the emergency department due to chest pain.

**How is the study done?**

If you decide to participate in the study, you will be interviewed by a computer-aided program on a tablet (iPad). The interview is extensive, and you will be asked about your symptoms, past and present illnesses, medications, heredity and social background.

The interview will take place during the waiting time at the emergency department and is estimated to take approximately one (1) hour. It will not affect the waiting time or delay any treatment you need. The staff at the emergency department will not have access to the answers you provide in the interview. In the emergency department, you will also meet a doctor who will collect a traditional medical interview.

The study does not involve any further visits or sampling. However, we will need your permission to access your electronic health record for one (1) year to come, to see what diagnosis you were given at this emergency department visit and at any additional medical encounters after one (1) year. We will also retrieve the results of the physical examination, ECG and blood samples from your current visit to the emergency department. Finally, we will also collect register data on medical visits from Stockholm County Council (SLL) and on sick leave from the Swedish Social Insurance Agency for (1) year to come.

You may later be asked to be interviewed or to fill out a questionnaire about your experience of using the system. You will then receive separate information and consent.

**Please note that if you experience new symptoms, recurrent or worsening chest pain, you should stop the interview immediately and call the staff at the emergency department.**

**What are the risks?**

No risks have been identified in previous studies when the computer program was used. It can be perceived as unpleasant to enter personal data on a tablet. There is a possibility that your visit to the emergency room will be prolonged. However, the risks of delaying time for necessary examination and treatment are very small as the computer-aided interview is normally done during waiting time and can be postponed and, for example, carried out at home.

**Are there any benefits?**

Through your participation in research studies, you contribute to new knowledge that other people can benefit from in the future.

**Data management and confidentiality**

Your answers and results will be processed so that unauthorized cannot take part of them. When stored, the information will be coded, which means that it cannot be directly traced to you as a person. The data will be stored on an encrypted computer (server) at Danderyd Hospital with a backup copy at Karolinska Institutet in Solna. The information cannot be accessed by unauthorized. When processing study data, your name and social security number are replaced by a code so that it is not possible to identify an individual. The research physician handles the code key in such a way that unauthorized access is prevented. Your data is processed in accordance with the EU General Data Protection Regulation (GDPR). Stockholm County Council (SLL) are responsible for your personal information.

The information may only be used in this study for which you have given your consent. It can only become relevant for new research projects after you have given a new consent and that approval has been given by the ethics review board.

**How do I get information about the results of the study?**

A description of this clinical study is available at http://www.clinicaltrials.gov. At most, it will contain a summary of the results. It can take several years before the research results are published. Your identity remains protected if any study results are published or made public.

**Insurance, compensation**

The Swedish patient injury insurance applies to the study in the same way as to the usual health care.

**Voluntary Participation**

Your participation in the study is entirely voluntary and you have the right to stop participating at any time, without giving an explanation. This will then not affect your treatment and continued care. The information you have already provided will be saved and deidentified. The study staff can choose to cancel your participation without your consent if he/she believes that your health condition justifies it.

You have the full right to withdraw your consent at any time and without explanation to store the information on the computer (server). The information will then be saved deidentified, but no new data will be collected.

**Responsible parties**

The legal entity and personal data controller are Stockholm County Council. If you have any questions or would like further information about the study, you can contact the people listed below.

Thomas Kahan Helge Brandberg X X

Professor, Senior consultant Doctoral student, Resident Study nurse

Department of Cardiology Department of Cardiology Department of Cardiology

Danderyd Hospital Danderyd Hospital Danderyd Hospital

08-123 XXX XX 08-123 XXX XX

# CLEOS-CPDS: Consent form

**Computerized history taking for management of chest pain patients in the emergency department**

I have received information about the above study. I have also had the opportunity to ask questions about the study and about my participation and I am satisfied with the answers and explanations given.

By signing this page, I agree to:

- Voluntarily participate in this study.
- The information I provide via the computer program and the information retrieved from my electronic health record at the current visit at the emergency department are handled according to the information in the patient information sheet.
- Information regarding diagnoses at new medical care contacts may be retrieved from my electronic health record for one (1) year ahead and handled according to the patient information sheet above.

I know that at any time and without giving any reason, I can withdraw my participation in the study without it affecting my future treatment and care.

______________________________ _____________________

Signature of patient Date (filled in by patient)

______________________________

Name of patient

I have explained the purpose and design of the study for the above mentioned patient. The patient will receive a copy of the patient information sheet and consent form if desired.

______________________________ _____________________

Signature of research staff Date (filled in by research staff)

______________________________

Name of research staff

# CLEOS-CPDS: Patientinformation

**Datoriserad anamnestagning vid omhändertagande av patienter med bröstsmärta på akutmottagningen**

**Bakgrund och syfte**

I Sverige söker fler än 200 000 personer/år akutmottagningen på grund av bröstsmärta. Orsaken till bröstsmärta kan vara allvarlig, men är ofta helt ofarlig. För att avgöra detta krävs undersökning, provtagning och läkarintervju. Trots detta kan det i vissa fall vara svårt att utesluta allvarlig bakomliggande orsak och ytterligare undersökningar eller observation på sjukhus kan då krävas. Dessutom har tidigare studier visat att patienter som söker på akuten för bröstsmärta, men som inte har en allvarlig sjukdom, ofta söker vård och är sjukskrivna i högre grad än friska.

Huvudsyftet med denna studie är att undersöka om en ingående intervju via en läsplatta (iPad) kan ge ett bättre underlag för läkaren än en traditionell intervju av läkaren på akutmottagningen för att avgöra om bröstsmärtan är ofarlig eller inte. Vi vill också undersöka om en sådan intervju kan förutsäga sjuklighet bland patienter som söker på akutmottagningen för bröstsmärta, men som inte har en allvarlig sjukdom. I framtiden hoppas vi kunna ge bättre behandling även för dessa patienter.

**Förfrågan om deltagande**

Du tillfrågas om att delta i studien eftersom Du har sökt akutmottagningen med bröstsmärta.

**Hur går studien till?**

Om Du bestämmer Dig för att delta i studien kommer Du att intervjuas av ett datorlett program på en läsplatta (iPad). Intervjun är ingående och Du kommer bland annat tillfrågas om Dina symtom, tidigare och nuvarande sjukdomar, läkemedel, ärftlighet och social bakgrund.

Intervjun kommer att ske under väntetiden på akutmottagningen och uppskattas ta ungefär en (1) timme. Den kommer inte påverka väntetiden eller fördröja den eventuella behandling Du behöver. Medarbetarna på akutmottagningen kommer inte ha tillgång till de svar Du anger i intervjun. Du kommer också alltid att träffa en läkare som kommer att göra en traditionell intervju på akutmottagningen

Studien innebär inga ytterligare besök eller provtagningar. Vi kommer dock att behöva Ditt tillstånd att titta i Din datorjournal under ett (1) år framåt för att se vilken diagnos som Du fick efter detta besök på akutmottagningen samt vid eventuella ytterligare sjukvårdskontakter efter ett (1) år. Vi kommer också att inhämta resultat av kroppsundersökningen, EKG och blodprover vid Ditt besök på akutmottagningen. Slutligen kommer vi också att inhämta registerdata om sjukvårdsbesök från Stockholms Läns Landsting (SLL) samt om sjukskrivning från Försäkringskassan under (1) år framåt.

Du kan senare komma att bli tillfrågad om att intervjuas eller att fylla i ett frågeformulär om Dina erfarenheter av att använda systemet. Du kommer då att få separat information och få lämna samtycke.

**Observera att om Du får nya symtom, återkommande eller förvärrad bröstsmärta ska Du avbryta intervjun omedelbart och tillkalla medarbetare på akutmottagningen.**

**Vilka är riskerna?**

Inga risker har påvisats vid tidigare studier då datorprogrammet använts. Det kan uppfattas som obehagligt att mata in personlig data i en läsplatta. Det finns en möjlighet att Ditt besök på akutmottagningen förlängs. Riskerna för fördröjning av tid till nödvändig undersökning och behandling är dock mycket liten eftersom den datoriserade intervjun normalt görs under väntetid och kan senareläggas och exempelvis genomföras i hemmet.

**Finns det några fördelar?**

Genom Ditt deltagande i studier bidrar Du till nya kunskaper som andra människor kan ha nytta av i framtiden.

**Hantering av data och sekretess**

Dina svar och resultat kommer att behandlas så att inte obehöriga kan ta del av dem. Uppgifterna kommer att förvaras kodade, vilket innebär att de inte direkt kan härledas till Dig som person. De kommer att lagras på en krypterad dator (server) på Danderyds sjukhus och en säkerhetskopia på Karolinska Institutet i Solna. Uppgifterna kan inte nås av obehöriga. Vid bearbetning av studiedata ersätts Ditt namn och personnummer av en kod så att det inte går att identifiera en enskild person. Studieläkaren förvarar kodnyckeln på sådant sätt att obehörig åtkomst förhindras. Din data bearbetas enligt EU:s dataskyddsförordning (GDPR). Ansvarig för Dina personuppgifter är Stockholms Läns Landsting (SLL).

Uppgifterna får endast användas i denna studie som Du gett samtycke till. De kan endast bli aktuella för ett nytt forskningsprojekt efter att Du lämnat ett nytt samtycke och att godkännande skett av etikprövningsnämnden.

**Hur får jag information om studiens resultat?**

En beskrivning av denna kliniska studie finns tillgänglig på http://www.clinicaltrials.gov. Som mest kommer den innehålla en sammanfattning av resultaten. Det kan dörja flera år innan forskningsresultaten publiceras. Din identitet förblir skyddad om några studieresultat publiceras eller offentliggörs.

**Försäkring, ersättning**

Den svenska patientskadeförsäkringen gäller för studien på samma sätt som för den vanliga hälso- och sjukvården.

**Frivillighet**

Ditt deltagande i studien är helt frivilligt och Du har rätt att när som helst, utan särskild förklaring avbryta Ditt deltagande. Detta kommer då inte att påverka Din behandling och fortsatta vård. De uppgifter Du redan hunnit lämna kommer då att sparas och avidentifieras. Studiepersonalen kan välja att avbryta Ditt deltagande utan Ditt samtycker om han/hon anser att Ditt hälsotillstånd motiverar det.

Du har full rätt att när som helst och utan förklaring ta tillbaka Ditt samtycke om att uppgifterna lagras på datorn (servern). Uppgifterna kommer då att sparas avidentifierat, men inga nya data kommer att samlas in.

**Ansvariga**

Forskningshuvudman och personuppgiftsansvarig är Stockholms Läns Landsting.

Om Du har några frågor eller önskar ytterligare information om studien kan Du vända Dig till de personer som anges nedan.

Thomas Kahan Helge Brandberg X X

Professor, överläkare Doktorand, ST-läkare Forskningssköterska

Hjärtkliniken Hjärtkliniken Hjärtkliniken

Danderyds sjukhus Danderyds sjukhus Danderyds sjukhus

08-123 XXX XX 08-123 XXX XX

# CLEOS-CPDS: Informerat samtycke

**Datoriserad anamnestagning vid omhändertagande av patienter med bröstsmärta på akutmottagningen**

Jag har fått information om ovanstående studie. Jag har också fått möjlighet att ställa frågor om studien och om mitt deltagande och jag är nöjd med de svar och förklaringar jag fått.

Genom att signera denna sida samtycker jag till att:

- Frivilligt delta i denna studie.
- De uppgifter jag lämnar via datorprogrammet och den information som hämtas från min datorjournal vid det aktuella besöket på akutmottagningen hanteras enligt uppgifterna i patientinformation.
- Uppgifter avseende diagnoser vid nya vårdkontakter får hämtas ur min datorjournal för ett (1) år framåt och hanteras enligt ovanstående patientinformation.

Jag vet att jag när som helst och utan att uppge något skäl kan avbyta mitt deltagande i studien utan att det påverkan min framtida behandling och vård.

______________________________ _____________________

Patientens underskrift Datum (fylls i av patienten)

______________________________

Patientens namn

Jag har förklarat studiens syfte och utformning för ovan nämnda patient. Patienten kommer att få en kopia av patientinformationen och samtycke om så önskas.

______________________________ _____________________

Studiepersonals underskrift Datum (fylls i av prövare)

______________________________

Studiepersonals namn
