# Supplementary material for: Use of Self-Reported Computerized Medical History Taking for Acute Chest Pain in the Emergency Department – the Clinical Expert Operating System Chest Pain Danderyd Study (CLEOS-CPDS): Prospective Cohort Study
Source: J Med Internet Res. 2021 Apr 27;23(4):e25493. doi: 10.2196/25493 (PMC8114166; doi:10.2196/25493)
Supplement: Multimedia Appendix 2 [file jmir_v23i4e25493_app2.docx]

| **Reasons** | **n (%)** |
| --- | --- |
| Language issues | 182 (18) |
| To tired | 158 (16) |
| Inability to use tablet | 152 (15) |
| No cause stated | 110 (11) |
| Lack of interest | 61 (6) |
| Admission/sent home | 57 (6) |
| Multiresistent bacteria | 37 (4) |
| Cognitive impairment/confusion/mental causes | 32 (3) |
| Visual impairment | 27 (3) |
| Physically affected/reduced general condition | 25 (3) |
| Pain | 25 (3) |
| Age | 20 (2) |
| Already participated | 18 (2) |
| Anxiety/stress | 15 (2) |
| Need of urgent medical care | 15 (2) |
| Asleep | 13 (1) |
| Do not want to participate in studies/share EHR | 13 (1) |
| Not found in the ED by research staff | 6 (1) |
| Busy with other things | 6 (1) |
| Other | 28 (3) |

EHR: electronic health record. ED: emergency department.
