# Supplementary material for: Use of Self-Reported Computerized Medical History Taking for Acute Chest Pain in the Emergency Department – the Clinical Expert Operating System Chest Pain Danderyd Study (CLEOS-CPDS): Prospective Cohort Study
Source: J Med Internet Res. 2021 Apr 27;23(4):e25493. doi: 10.2196/25493 (PMC8114166; doi:10.2196/25493)
Supplement: Multimedia Appendix 3 [file jmir_v23i4e25493_app3.docx]

|  | Max. duration (min) | **n,** crude | Fraction, % | **n,** with all pauses > 2  excluded | Fraction, % |
| --- | --- | --- | --- | --- | --- |
| **All, n=500** | |  |  |  |  |
|  | 3 | 4 | 1 | 10 | 2 |
|  | 5 | 11 | 2 | 22 | 4 |
|  | 10 | 32 | 6 | 51 | 10 |
|  | 15 | 44 | 9 | 81 | 16 |
|  | 20 | 68 | 14 | 105 | 21 |
|  | 30 | 106 | 21 | 167 | 33 |
|  | 45 | 178 | 36 | 257 | 51 |
|  | 60 | 248 | 50 | 350 | 70 |
|  | 75 | 319 | 64 | 423 | 85 |
|  | 90 | 370 | 74 | 468 | 94 |
|  | 105 | 410 | 82 | 488 | 98 |
|  | 120 | 442 | 88 | 495 | 99 |
|  | > 120 | 58 | 12 | 5 | 1 |
| **Women, n=213** | |  |  |  |  |
|  | 3 | 4 | 2 | 5 | 2 |
|  | 5 | 6 | 3 | 12 | 6 |
|  | 10 | 18 | 9 | 27 | 13 |
|  | 15 | 24 | 11 | 41 | 19 |
|  | 20 | 35 | 16 | 52 | 24 |
|  | 30 | 49 | 23 | 78 | 37 |
|  | 45 | 72 | 34 | 109 | 51 |
|  | 60 | 103 | 48 | 152 | 71 |
|  | 75 | 131 | 62 | 183 | 86 |
|  | 90 | 155 | 73 | 199 | 93 |
|  | 105 | 169 | 79 | 208 | 97 |
|  | 120 | 184 | 86 | 210 | 99 |
|  | > 120 | 29 | 14 | 3 | 1 |
| **Men, n=287** | |  |  |  |  |
|  | 3 | 0 | 0 | 5 | 2 |
|  | 5 | 5 | 2 | 10 | 4 |
|  | 10 | 14 | 5 | 24 | 8 |
|  | 15 | 20 | 7 | 40 | 14 |
|  | 20 | 33 | 12 | 53 | 19 |
|  | 30 | 57 | 20 | 89 | 31 |
|  | 45 | 106 | 37 | 148 | 52 |
|  | 60 | 145 | 51 | 198 | 69 |
|  | 75 | 188 | 66 | 240 | 84 |
|  | 90 | 215 | 75 | 269 | 94 |
|  | 105 | 241 | 84 | 280 | 98 |
|  | 120 | 258 | 90 | 285 | 99 |
|  | > 120 | 29 | 10 | 2 | 1 |
