# Supplementary figures and images for: Use of Self-Reported Computerized Medical History Taking for Acute Chest Pain in the Emergency Department – the Clinical Expert Operating System Chest Pain Danderyd Study (CLEOS-CPDS): Prospective Cohort Study
Source: J Med Internet Res. 2021 Apr 27;23(4):e25493. doi: 10.2196/25493 (PMC8114166; doi:10.2196/25493)

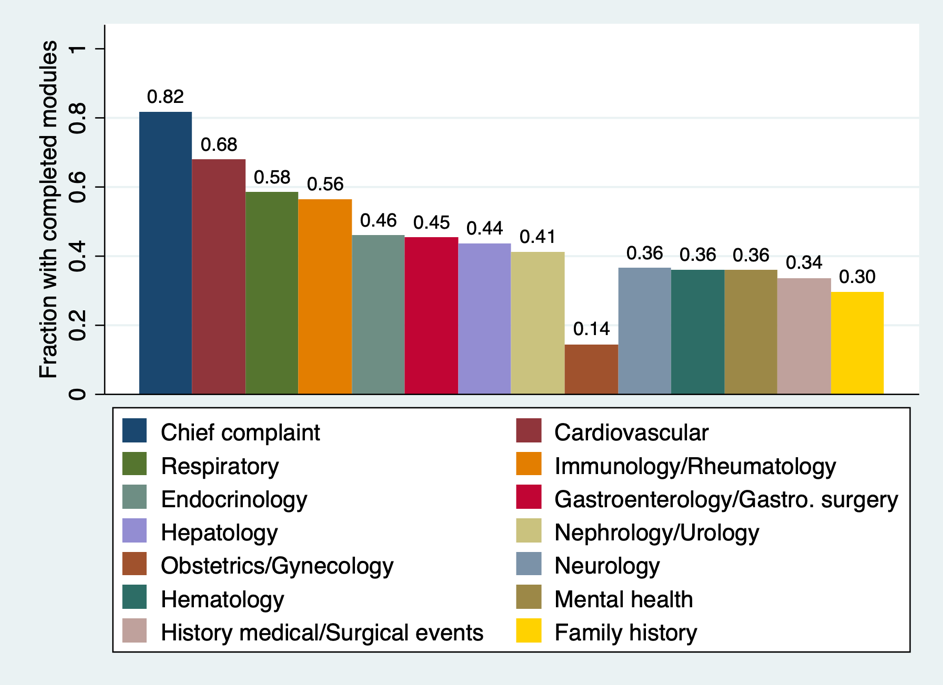

Supplement: Multimedia Appendix 4 [file jmir_v23i4e25493_app4.png]
