# Supplementary material for: Use of Self-Reported Computerized Medical History Taking for Acute Chest Pain in the Emergency Department – the Clinical Expert Operating System Chest Pain Danderyd Study (CLEOS-CPDS): Prospective Cohort Study
Source: J Med Internet Res. 2021 Apr 27;23(4):e25493. doi: 10.2196/25493 (PMC8114166; doi:10.2196/25493)
Supplement: Multimedia Appendix 5 [file jmir_v23i4e25493_app5.docx]

| **Interview duration (min)** | n | Proportion, % | Pauses | Average number of pauses > 2 | Mean paus duration (min:s) |
| --- | --- | --- | --- | --- | --- |
| <15 | 44 | 9 | 19 | 0.4 | 04:41 |
| 15-29 | 62 | 12 | 67 | 1.1 | 04:36 |
| 30-44 | 72 | 14 | 101 | 1.4 | 05:55 |
| 45-59 | 70 | 14 | 143 | 2.0 | 06:29 |
| 60-74 | 71 | 14 | 182 | 2.6 | 07:07 |
| 75-89 | 51 | 10 | 180 | 3.5 | 07:31 |
| 90-104 | 40 | 8 | 144 | 3.6 | 07:34 |
| 105-119 | 32 | 6 | 177 | 5.5 | 07:55 |
| >120 | 58 | 12 | 391 | 6.7 | 18:38 |
